# Pre-pregnancy cardiovascular risk factors and racial disparities in birth outcomes: the Bogalusa Heart Study

**DOI:** 10.1186/s12884-018-1959-y

**Published:** 2018-08-20

**Authors:** Emily W. Harville, Leann Myers, Tian Shu, Maeve E. Wallace, Lydia A. Bazzano

**Affiliations:** 1 Department of Epidemiology, Tulane School of Public Health and Tropical Medicine, 1440 Canal St. Ste. 2001, #8318, New Orleans, LA 70112-2715 USA; 2 Department of Global Biostatistics and Data Science, Tulane School of Public Health and Tropical Medicine, New Orleans, LA USA; 3 Department of Global Community Health and Behavioral Sciences, Tulane School of Public Health and Tropical Medicine, New Orleans, LA USA

**Keywords:** Birth weight, low, Premature birth, Infant, small for gestational age, Cholesterol, Glucose, Continental population groups

## Abstract

**Background:**

Racial disparities in birth outcomes are mirrored in cardiovascular health. Recently there have been calls for more attention to preconception and interconceptional health in order to improve birth outcomes, including as a strategy to reduce black-white disparities.

**Methods:**

As part of a larger study of cardiovascular and reproductive health (“Bogalusa Babies”), female participants were linked to their children’s birth certificates for Louisiana, Mississippi, and Texas births from 1982 to 2009. Three thousand and ninety-five women were linked to birth certificate data. Birth outcomes were defined as low birthweight (LBW) birthweight < 2500 g; preterm birth (PTB), > 3 weeks early; small for gestational age (SGA), <10th percentile for gestational age (percentiles based on study population); large for gestational age (LGA) >90th percentile for gestational age]. Cardiovascular measures (blood pressure, lipids, glucose, insulin) at the visit closest in time but prior to the pregnancy was examined as predictors of birth outcomes using logistic models adjusted for covariates.

**Results:**

Only a few cardiovascular risk factors were associated with birth outcomes. Triglycerides were associated with higher risk of LBW among whites (aOR 1.05, 95% 1.01–1.10). Higher glucose was associated with a reduction in risk of SGA for black women (aOR 0.85, 95% CI 0.76–0.95), but not whites (p for interaction = 0.02). Clear racial disparities were found, but they were reduced modestly (LBW/SGA) or not at all (PTB/LGA) after CVD risk factors were adjusted for.

**Conclusions:**

This analysis does not provide evidence for preconception cardiovascular risk being a strong contributor to racial disparities.

**Electronic supplementary material:**

The online version of this article (10.1186/s12884-018-1959-y) contains supplementary material, which is available to authorized users.

## Background

In the United States, blacks have roughly double the risk of infant mortality of other ethnic groups [1], due largely to preterm birth and fetal growth restriction. This disparity persists across populations with comparable access to health care and in otherwise low-risk groups, such as military populations, those with health insurance, and women with higher education and low initial medical risk [2–4]. Disparities in birth outcomes are mirrored in cardiovascular health. Black women have higher cardiovascular risk than other racial/ethnic groups [5], including higher blood pressure and diabetes [6]. In NHANES, black women of childbearing age had higher systolic blood pressure, diastolic blood pressure, and glycated hemoglobin than other groups [7].

Recently there have been calls for more attention to preconception and interconceptional health in order to improve birth outcomes [8–11], including as a strategy to reduce black-white disparities [12, 13], in part because black women have an increased risk of preconception hypertension and diabetes [14]. Women with chronic hypertension who become pregnant are at higher risk of preterm birth and small-for-gestational-age [15, 16], while women with diabetes who become pregnant are at higher risk of preterm birth [16–18] and large-for-gestational-age/macrosomia [19]. Some studies indicate that preconception cardiovascular health outside clinical disease also impacts pregnancy health: higher preconception blood pressure has been associated with lower birth weight [20], while both low and high cholesterol have been associated with poor pregnancy outcome [20, 21]. In addition, preconception glucose levels are associated with increased birthweight [20]. In our analysis of the Cardiovascular Risk in Young Finns Study [22], higher pre-pregnancy triglycerides were associated with a higher risk of hypertensive disorders, pre-eclampsia, and gestational diabetes, while higher blood pressure was associated with preterm birth and small-for-gestational-age.

Still, few studies have examined preconception cardiovascular health and birth outcomes [21], and some of the largest are limited to Scandinavian populations [20, 22]. While some studies have examined clinical comorbidities as predictors of birth outcomes in black women [23], few have assessed subclinical measures. In this study, we examine how pre-pregnancy cardiovascular risk factors are associated with birth outcomes in the Bogalusa Heart Study, a biracial study of cardiovascular health. The research questions are a) are pre-pregnancy cardiovascular risk factors associated with birth outcomes in this cohort; b) do any associations between these risk factors and the outcomes differ between African-American and white women? Based on the results of the analyses of those questions, we then examined whether pre-pregnancy cardiovascular health contribute to racial disparities in birth outcomes.

## Methods

The Bogalusa Heart Study is a long-running study of childhood, adolescent, and now adult cardiovascular health, founded by Dr. Gerald Berenson in 1973 [24]. Participants were initially recruited from schools in the Bogalusa, Louisiana, area at ages 3–18. Over time, additional waves of data collection were performed, adding additional participants up to adulthood. Currently, participants are largely in their 40s through 60s, and follow-up for cardiovascular and early aging parameters continues. The average age at first visit was 8.5 for black women and 8.6 for white women, *p* = 0.32.

As part of a larger study of cardiovascular and reproductive health (“Bogalusa Babies”), female participants were linked to their children’s birth certificates for Louisiana, Mississippi, and Texas births from 1982 to 2009. The 1982–1989 records contained fewer variables for linkage than later years; observations that matched on four variables (year of birth, last name, Soundex code for last name, and race) were confirmed by visual comparison of addresses. From 1990 to 2009, a three stage linkage process was used, including deterministic record linkage based on maternal social security number (SSN), and probabilistic linkage when SSN was unavailable.

Five thousand, nine hundred and- ten women/girls participated in BHS at least once during the study. Of those, 3263 were linked to birth certificate data, representing a linkage of approximately 65% of the likely births (as approximately 10–15% of women do not give birth). Participants linked to birth records were more likely to be black (37% vs. 35%), had more study visits, had a younger average age at first visit (8.8 vs. 10.5), were less likely to smoke (35% vs. 38%), and had statistically lower mean BMIs (19.9 vs. 20.3) and blood pressure (101.8 vs. 102.8) (Harville et al., under review). Among white women, those whose parents had a high school education, rather than more or less education, were most likely to be linked, while among black women, likelihood of linkage fell with increased parental education. Among the smaller group with information about adult education, higher education was associated with increased likelihood of linkage among both black and white women. Three thousand and ninety-five women had at least one screening as a child, which occurred prior to the first pregnancy in the dataset. Two thousand, seven hundred and sixty-three screenings included a fasting blood samples; 2691 of these had data for analysis of lipids, insulin, and glucose. (More detail on missing data for covariates or individual risk factors is found in the tables). If a woman had multiple pregnancies, the first linked pregnancy was used, and analysis was limited to singleton births.

### Exposures

Blood pressure levels were measured on the right arm of subjects in a relaxed, sitting position by two trained observers (3 replicates each). Systolic blood pressure and diastolic blood pressure were recorded using a mercury sphygmomanometer. The fifth Korotkoff phase was used for diastolic blood pressure. The mean values of the readings were used for analysis.

Fasting blood samples were drawn for lipids and glucose analysis. Prior to 1987, serum total cholesterol and triglyceride levels were determined by a Technicon AutoAnalyzer II (Technicon Instrument, Tarrytown, NY). From 1987 to 1996, cholesterol and triglyceride levels were determined by enzymatic procedures on the Abbott VP instrument (Abbott Laboratories, Chicago, IL) and on the Hitachi 902 Automatic Analyzer (Roche Diagnostics, Indianapolis, IN) after 1996. Serum lipoprotein cholesterols were analyzed by using a combination of heparin-calcium precipitation and agar-agarose gel electrophoresis procedures. Both chemical and enzymatic procedures met the performance requirements of the Lipid Standardization Program of the Centers for Disease Control and Prevention, which has routinely monitored the precision and accuracy of cholesterol and triglyceride measurements since 1973. Measurements on CDC-assigned quality control samples showed no consistent bias over time within or between surveys.

From 1981 to 1991, plasma glucose was measured by a glucose oxidase method using a Beckman Glucose Analyzer (Beckman Instruments, Palo Alto, CA). Since then, it has been measured enzymatically as part of a multichemistry (SMA20) profile.

Measurements were made by laboratory technicians blinded to participants’ risk factors. Ten percent blind duplicate samples are selected, prior to blood drawing. The intraclass correlation coefficient for blind duplicate samples ranged from 0.92 for glucose to 0.99 for total cholesterol.

Birth outcomes were taken from measures on the birth certificates. Low birthweight (LBW) was defined as birthweight < 2500 g, preterm birth (PTB) as birth < 37 weeks (obstetric estimate, when available); small for gestational age (SGA) was defined as <10th percentile for gestational age (percentiles based on study population) and large for gestational age (LGA) as >90th percentile for gestational age. Report of both birthweight and gestational age on birth certificates have been found to be highly accurate [25, 26]. A secondary analysis looked at birthweight as a continuous outcome.

Covariates were chosen a priori based on factors known to be associated with exposure and outcome [27, 28]. Race was recorded as white or black at the initial BHS visit. Later follow-ups and birth certificate data with more extensive options indicated that this was consistent with the racial/ethnic self-categorization of almost all participants (< 10 women had Hispanic ethnicity marked on the birth certificate).

Age at pregnancy was calculated from participant’s date of birth. Data on parity, weight gain, maternal education, and smoking were taken from the birth certificate data. BMI at time of visit was calculated from measured height and weight: participants were measured in duplicate and the average of the measures was used. Adequacy of prenatal care was assessed using the Kotelchuck index [29], and categorized as inadequate, intermediate, adequate, and adequate plus.

### Analysis

Cardiovascular measures at the visit closest in time but prior to the pregnancy (based on an estimated last menstrual period 40 weeks before birthdate) were examined as predictors. Logistic regression was used for dichotomous measures. Models were adjusted for variables that were associated with the outcome at *p* < 0.20: these varied by outcome but included age, race, smoking, parity, maternal education, adequacy of prenatal care, BMI at time of visit, age at time of Bogalusa screening, and years between Bogalusa screening and birth. Due to the strong correlation between BMI and cardiovascular risk factors, results are presented adjusted for BMI alone, then with adjustment for all factors. In addition to stratified models, combined models were run with an interaction term for race to examine differences between white and black participants. Results are presented stratified by race. As some studies have found non-linear associations between cardiovascular risk factors and birth outcomes [21], quadratic terms were also examined. Quadratic BMI was examined as a potential confounder, to assess possible non-linear confounding by BMI, but this term was neither statistically predictive nor did it change the effect estimate. We attempted to determine whether results were different when limited to those whose study visit was closer in time to the pregnancy, but sample size was relatively small (*n* = 586, 217 black, 369 white for those with a measure within 5 years of pregnancy) and results became too imprecise to judge whether this group differed from the main sample.

Racial disparities in birth outcomes were examined, first, unadjusted, then adjusted for the same confounders identified in the previous analysis. Finally, results were presented adjusted for these confounders as well as cardiovascular risk factors identified as predictors of birth outcomes in this or other analyses, including quadratic associations. As the associations found in this analysis were relatively weak, no formal mediation analysis was performed.

Participants’ parents provided informed consent for child visits and adult participants provided their own informed consent for BHS measures. The Institutional Review Boards (IRB) of Tulane University (IRB ID#256406), the State Department of Health and Hospitals of Louisiana, and the Texas Department of State Health Services approved the linkage protocol (Mississippi deferred to the Tulane IRB). The linkage was conducted under a waiver of consent, as it was deemed minimal risk and infeasible without the waiver.

## Results

The study population was approximately one-third black and two-thirds white (Table 1). The majority of the linked pregnancies were first births, although 30% were not. Smoking during pregnancy was listed for 13%. The average age at visit prior to pregnancy was 13 yrs. (range 4–38) and average time between study visit and pregnancy was 10.8 years (range 0.8–33). Mean and median age at included pregnancy were 24.2 and 23.0.

**Table 1:** Participants in the Bogalusa Heart Study linked to birth certificates (*N* = 3095)

	Overall study population		Black (*n* = 1139)		White (*n* = 1956)		*p*-value
	N	%	N	%	N	%	
parity							< 0.01
1	2116	68.4	760	65.5	1384	70.3	
2	630	20.4	229	19.7	410	20.8	
3	243	7.9	106	9.1	137	7.0	
4+	104	3.3	66	5.7	38	1.9	
education							< 0.01
< high school	780	25.2	343	29.6	448	22.7	
high school graduate	1190	38.5	474	40.9	732	37.2	
some college	575	18.6	219	18.9	361	18.3	
college & beyond	547	17.7	124	10.7	429	21.8	
married							< 0.01
yes	1638	53.0	256	22.1	1398	71.0	
no	1452	47.0	903	77.9	571	29.0	
smoking							< 0.01
yes	376	12.8	380	12.8	328	17.4	
no	2566	87.2	2595	87.2	1559	82.6	
	N	mean + SD	N	mean + SD	N	mean + SD	
age at screening	3095	13.0 + 6.7	1139	12.5 + 6.4	1956	13.3 + 6.8	< 0.01
time from screening (yrs)	3095	10.8 + 5.8	1139	10.1 + 5.3	1956	11.1 + 6.0	< 0.01
maternal age	3095	24.2 + 5.4	1139	23.0 + 5.4	1956	24.9 + 5.3	< 0.01
pregnancy weight gain (lbs)	2645	30.8 + 13.6	930	29.0 + 13.8	1715	31.7 + 13.4	< 0.01
BMI	3089	20.1 + 5.5	1136	20.4 + 5.9	1953	20.0 + 5.2	0.08
systolic blood pressure	3090	102.0 + 10.5	1136	102.4 + 11.1	1954	101.8 + 10.1	0.11
diastolic blood pressure	3090	63.1 + 9.7	1136	62.7 + 10.5	1954	63.3 + 9.2	0.13
fasted only:							
cholesterol	2691	170.4 + 30.9	976	173.2 + 31.7	1715	168.7 + 30.3	< 0.01
triglycerides	2691	77.2 + 45.9	976	66.1 + 29.9	1715	83.5 + 51.8	< 0.01
insulin	2098	11.2 + 8.4	777	12.4 + 10.6	1321	10.5 + 6.6	< 0.01
glucose	2447	78.8 + 16.0	888	77.5 + 17.7	1559	79.4 + 14.9	0.01
LDL-C	2688	103.0 + 28.1	974	103.9 + 28.5	1714	102.4 + 27.9	0.21
HDL-C	2690	56.7 + 16.4	975	60.4 + 16.0	1715	54.5 + 16.3	< 0.01
	N	%	N	%	N	%	
Low birthweight (full-term only)	91	3.3	52	5.2	39	2.2	< 0.01
Preterm birth	297	9.6	131	11.5	166	8.5	< 0.01
Small for gestational age	353	11.4	182	16.0	171	8.7	< 0.01
Large for gestational age	298	9.6	64	5.6	234	12.0	< 0.01

Only a few cardiovascular risk factors were associated with birth outcomes. Triglycerides were associated with higher risk of LBW among whites (aOR 1.05, 95% 1.01–1.10; Table 2); the effect estimate was very similar although not statistically significant for blacks (1.07, 0.97–1.19). Higher glucose was associated with a reduction in risk of SGA for black women (aOR 0.86, 95% CI 0.79–0.95; Table 3), but not whites (p for interaction = 0.02). No associations were found with PTB (Table 4) or LGA (Table 5). Results were similar for birthweight as a continuous outcome (Additional file 1: Table S1).

**Table 2:** Relationship between preconception cardiovascular risk factors and low birthweight

	White						Black						p for race^*^ risk factor interaction		
	unadjusted		adjusted^¶^		adjusted^**^		unadjusted		adjusted^¶^	adjusted^**^			un-adjusted	adjusted^¶^	adjusted^**^
	OR	95% CI	OR	95% CI	OR	95% CI	OR	95% CI	OR	95% CI	OR	95% CI			
systolic BP	1.17	0.86–1.60	0.91	0.78–1.07	1.41	0.93–2.13	1.02	0.79–1.31	1.02	0.78–1.32	0.93	0.65–1.34	0.49	0.51	0.29
diastolic BP	1.01	0.72–1.43	0.94	0.79–1.12	0.99	0.60–1.63	0.95	0.73–1.24	0.98	0.75–1.29	0.79	0.54–1.15	0.77	0.76	0.54
cholesterol^*^	0.97	0.87-1.09	1.01	0.96–1.05	1.03	0.90–1.18	1.00	0.91–1.11	1.00	0.91–1.09	1.00	0.88–1.13	0.68	0.69	0.74
triglycerides^*^	1.03	0.99-1.07	1.01	0.98–1.03	1.05	1.01–1.10	1.05	0.97–1.15	0.98	0.88–1.09	1.07	0.97–1.19	0.62	0.42	0.56
LDL^*^	0.95	0.83-1.08	0.99	0.94–1.04	1.00	0.85–1.17	0.97	0.87–1.09	1.01	0.91–1.11	0.94	0.81–1.09	0.74	0.74	0.43
HDL^*^	1.01	0.82-1.24	1.05	0.96–1.15	0.94	0.72–1.23	1.10	0.91–1.32	0.95	0.79–1.14	1.15	0.91–1.46	0.54	0.53	0.18
glucose^*^	1.03	0.80-1.34	1.01	0.92–1.11	1.16	0.78–1.73	0.90	0.77–1.06	1.05	0.89–1.23	0.91	0.72–1.16	0.39	0.38	0.46
insulin^*^	1.03	0.59-1.80	0.92	0.68–1.23	1.39	0.79–2.44	1.13	0.90–1.41	0.77	0.44–1.34	1.10	0.87–1.40	0.78	0.88	0.91

^*^fasted

^¶^adjusted for BMI at last screening

^**^adjusted models include risk factor, cigarettes, Kotelchuck index, maternal education, parity, mother’s age at child’s birth, year of birth, BMI at last screening, and time (in years) between screening and birth

**Table 3:** Relationship between preconception cardiovascular risk factors and small-for-gestational-age

	white						black						p for race^*^RF interaction		
	unadjusted		adjusted^¶^		adjusted^‡^		unadjusted		adjusted^¶^		adjusted^‡^		unadjusted	adjusted^¶^	adjusted^‡^
	OR	95% CI	OR	95% CI	OR	95% CI	OR	95% CI	OR	95% CI	OR	95% CI			
systolic BP	0.97	0.83–1.14	1.06	0.88–1.27	1.08	0.88–1.32	0.96	0.83–1.11	0.99	0.84–1.17	1.03	0.85–1.25	0.87	0.88	0.51
diastolic BP	0.85	0.72–1.01	0.89	0.73–1.08	0.84	0.66–1.06	0.93	0.80–1.09	0.96	0.80–1.13	1.02	0.84–1.26	0.44	0.45	0.69
cholesterol^*^	1.00	0.95–1.06	1.01	0.95–1.07	1.01	0.95–1.08	0.96	0.91–1.02	0.96	0.91–1.02	0.96	0.90–1.02	0.30	0.28	0.15
triglycerides^*^	1.00	0.96–1.03	1.00	0.97–1.04	0.99	0.95–1.04	1.02	0.97–1.08	1.04	0.98–1.11	1.04	0.97–1.11	0.32	0.24	0.32
LDL^*^	1.00	0.94–1.06	1.00	0.94–1.07	0.99	0.92–1.06	0.94	0.89–1.01	0.96	0.90–1.02	0.94	0.87–1.01	0.36	0.35	0.22
HDL^*^	1.04	0.93–1.15	1.02	0.92–1.14	1.11	0.98–1.26	0.97	0.87–1.08	0.95	0.85–1.06	0.99	0.87–1.12	0.33	0.33	0.28
glucose^*^	1.05	0.92–1.20	1.04	0.91–1.19	1.11	0.95–1.29	0.86	0.79–0.95	0.85	0.77–0.94	0.85	0.76–0.95	0.02	0.02	0.02
insulin^*^	0.94	0.69–1.27	1.02	0.74–1.41	0.99	0.68–1.43	1.06	0.90–1.25	1.08	0.91–1.28	1.06	0.89–1.27	0.48	0.59	0.55

* fasted

^¶^adjusted for BMI

^‡^adjusted for smoking, Kotelchuck index, maternal education, parity, married, maternal age, time since screening, BMI

**Table 4:** Relationship between preconception cardiovascular risk factors and preterm birth

	white						black						p for race^*^ risk factor interaction		
	unadjusted		adjusted^¶^		adjusted^†^		unadjusted		adjusted^¶^		adjusted^†^				
	OR	95% CI	OR	95% CI	OR	95% CI	OR	95% CI	OR	95% CI	OR	95% CI	Unadjusted	adjusted^¶^	adjusted^†^
systolic BP	0.97	0.83–1.13	1.09	0.91–1.31	1.18	0.96–1.45	0.90	0.76–1.06	1.01	0.83–1.22	1.09	0.87–1.35	0.56	0.54	0.48
diastolic BP	0.90	0.76–1.07	1.00	0.82–1.21	1.21	0.97–1.52	0.97	0.81–1.15	1.10	0.90–1.34	1.21	0.96–1.53	0.57	0.60	0.89
Cholesterol^*^	1.01	0.95–1.07	1.02	0.97–1.08	1.01	0.95–1.08	0.94	0.88–1.01	0.95	0.89–1.01	0.95	0.89–1.03	0.12	0.10	0.25
Triglyce-rides^*^	0.95	0.90–1.00	0.96	0.91–1.01	0.98	0.93–1.03	0.97	0.91–1.04	0.99	0.92–1.07	0.99	0.91–1.08	0.59	0.50	0.82
LDL^*^	1.00	0.94–1.07	1.02	0.96–1.08	1.01	0.94–1.08	0.93	0.87–1.00	0.94	0.87–1.02	0.92	0.85–1.00	0.14	0.12	0.14
HDL^*^	1.10	1.00–1.22	1.08	0.97–1.20	1.03	0.92–1.17	1.00	0.89–1.13	0.98	0.86–1.11	1.08	0.93–1.24	0.23	0.25	0.89
glucose^*^	0.98	0.87–1.10	0.96	0.85–1.09	0.94	0.82–1.07	0.89	0.80–0.99	0.86	0.77–0.96	0.86	0.75–0.99	0.21	0.19	0.15
insulin^*^	0.93	0.68–1.28	1.08	0.80–1.48	0.89	0.58–1.38	0.82	0.60–1.13	0.88	0.64–1.22	0.96	0.70–1.33	0.60	0.51	0.94

* fasted

^¶^adjusted for BMI

^†^adjusted for smoking, Kotelchuck index, married, maternal education, time since screening, BMI, year of birth

**Table 5:** Relationship between preconception cardiovascular risk factors and large-for-gestational-age

	white						black						unadjusted	adjusted	adjusted^§^
	unadjusted		adjusted^¶^		adjusted^§^		unadjusted		adjusted^¶^		adjusted^§^				
	OR	95% CI	OR	95% CI	OR	95% CI	OR	95% CI	OR	95% CI	OR	95% CI			
systolic BP	1.04	0.91–1.19	0.91	0.78–1.07	0.86	0.72–1.02	0.99	0.79–1.24	1.02	0.78–1.32	0.97	0.71–1.31	0.72	0.69	0.66
diastolic BP	1.07	0.92–1.25	0.94	0.79–1.12	0.91	0.75–1.10	0.96	0.76–1.22	0.98	0.75–1.29	0.96	0.70–1.31	0.45	0.48	0.49
cholesterol^*^	1.02	0.98–1.07	1.01	0.96–1.05	1.02	0.97–1.08	1.00	0.91–1.09	1.00	0.91–1.09	1.00	0.91–1.10	0.69	0.71	0.57
triglycerides^*^	1.02	0.99–1.04	1.01	0.98–1.03	1.00	0.98–1.03	0.97	0.88–1.08	0.98	0.88–1.09	1.00	0.89–1.11	0.43	0.33	0.48
LDL^*^	1.01	0.96–1.06	0.99	0.94–1.04	1.01	0.95–1.06	1.00	0.91–1.11	1.01	0.91–1.11	1.02	0.92–1.14	0.96	0.96	0.93
HDL^*^	1.01	0.93–1.11	1.05	0.96–1.15	1.05	0.95–1.15	0.96	0.80–1.15	0.95	0.79–1.14	0.88	0.71–1.09	0.61	0.58	0.22
glucose^*^	0.99	0.89–1.09	1.01	0.92–1.11	0.97	0.88–1.07	1.05	0.89–1.23	1.05	0.89–1.23	1.07	0.90–1.26	0.51	0.59	0.30
insulin^*^	1.10	0.88–1.38	0.92	0.68–1.23	0.85	0.62–1.16	0.86	0.55–1.34	0.77	0.44–1.34	0.74	0.42–1.31	0.32	0.35	0.40

* fasted

^¶^adjusted for BMI

^§^adjusted for smoking, parity, time since screening, BMI, maternal age

A quadratic association was found with systolic blood pressure, indicating higher risk for LBW and SGA at lower and higher levels (*p* < 0.05 for quadratic term).

Finally, we directly assessed whether cardiovascular risk might contribute to racial disparities. Racial disparities were clearly present in the sample: black women were at increased risk for LBW (Table 6; adjusted for non-cardiovascular risk factors, OR 3.84, 95% CI 2.07–7.12), PTB (1.66, 1.24–2.23), and SGA (2.18, 1.61–2.96), and reduced risk for LGA (0.35, 0.25–0.48). Further adjustment for cardiovascular risk factors attenuated the LBW and SGA estimates by approximately 10%.

**Table 6:** Racial disparities in birth outcomes and cardiovascular risk factors

	unadjusted		adjusted		additionally adjusted for cardiovascular risk factors	
	OR	95% CI	OR	95% CI	OR	95% CI
Low birthweight	2.44	1.69–3.73	3.84^a^	2.07-7.12	3.32^e^	1.52-7.21
Preterm birth	1.40	1.10–1.79	1.66^b^	1.24–2.23	1.65^f^	1.19-2.30
Small-for-gestational-age	1.99	1.59–2.48	2.18^c^	1.61-2.96	1.95^g^	1.37-2.76
Large-for-gestational-age	0.44	0.33–0.58	0.35^d^	0.25-0.48	0.32^h^	0.22-0.45

Adjusted for:

^a^(same adjustments as previous tables) smoking, Kotelchuck index, married, maternal education, time since screening, BMI, year of birth

^b^smoking, Kotelchuck index, maternal education, parity, maternal age, year of birth, time since screening, BMI

^c^smoking, Kotelchuck index, maternal education, parity, married, maternal age, time since screening, BMI

^d^smoking, parity, time since screening, BMI, maternal age

^e^previous column + triglycerides, insulin, systolic blood pressure, systolic blood pressure-squared

^f^previous column + glucose, LDL

^g^previous column + glucose, systolic blood pressure, systolic blood pressure-squared

^h^previous column + glucose

## Discussion

In this study, we attempted to determine the relationship between pre-pregnancy cardiovascular risk factors and disparities in birth outcomes. Although the expected racial disparities in cardiovascular risk factors and birth outcomes were found, there were only limited relationships among those factors. We found some evidence of inverse risk for associations between lipids and birthweight, largely among whites; previous studies have tended to find positive associations between pre-pregnancy lipids and birthweight [20]. Although not statistically significant, the size of the effect estimate for systolic blood pressure and birthweight was also consistent with previous studies in Scandinavian populations [20, 22]. Although previous studies of pre-pregnancy blood pressure have not found quadratic associations, such associations have been found in studies of blood pressure during pregnancy [30]. When associations were seen in black women, they tended to be protective, with higher glucose being associated with a reduced risk of LBW and SGA, consistent with previous studies [20]. A previous study of preterm birth found increased risk with both low and high levels of pre-pregnancy cholesterol, but did not find racial differences [21].

Preconception cardiovascular risk could lead to poorer birth outcomes by affecting placentation in the first trimester [21, 31, 32], increasing inflammation [33], or producing epigenetic changes that carry into pregnancy [34, 35]. It could also increase the risk of complications such as pre-eclampsia and gestational diabetes [36–38]; vital records data often have limited validity for those complications and record them in a manner problematic for this type of analysis (e.g., grouping pre-pregnancy and gestational diabetes) [25, 26]. Racial disparities are also apparent in particularly pregnancy-induced hypertension and pre-eclampsia, and thus these complications could mediate an effect of cardiovascular risk on birth outcomes [39–41].

Strengths of the study include the prospective data collection; well-characterized cardiovascular risk factors; a fairly large, biracial cohort; and linkage to participants regardless of later participation. Limitations include the variation in time between the pregnancy and the measure, and the lack of information on cardiovascular risk during the pregnancy, which would assist in determining whether preconception cardiovascular risk provides any additional information beyond that determined during pregnancy. In addition, we are limited to those who were able to be linked to vital statistics data. Comparisons of those who are linked and who were not does not point to a strongly high- or low-risk profile in those who were excluded; still, this set of potential participants represents a group that has the potential to change the results if they had been able to be included. On balance, there are indicators that both high- and low-risk women may have been less likely to be included; this may have reduced the variability in the sample and thus limited our ability to find differences.

Measurement error per se – of the included measures - should be limited. BHS has rigorous quality control methods, including measurement in duplicate, throughout. Birth certificate data is generally good quality for preterm birth and low birthweight [42], although gestational age measurements may be of limited quality for the oldest women, as ultrasound dating was less consistent during the time period of their pregnancies [43]. However, measurement error still exists in the sense that a single risk factor measurement is representing a large time period, and the measurements were not taken at the same time point for every woman, compounding the degree of error. This error would tend to bias the results towards the null, and it is possible that we would see a greater effect with measurements nearer in time to the pregnancy; our sample size for this analysis was too small to provide much information on this point. Measurement of non-cardiovascular covariate data is potentially more problematic. Tobacco use tends to be underreported on birth certificates [44]. Adjustment for covariates is fairly limited, although the strongest risk factors that were likely to be associated with exposure and outcomes were included. Overall, residual confounding would likely bias away from the null. Similarly, if cardiovascular risk factors are acting as confounders of the race-birth outcome relationship, imperfect measurement could be leading to residual confounding and preventing full adjustment for those factors, suggesting that better measures would more fully attenuate the relationships. Although prior preterm birth and low birthweight are associated with the outcome, in most cases the cardiovascular measure would have occurred prior to that birth as well, so it is unlikely that adjustment for this risk factor would be valid [45].

## Conclusions

Mechanisms underlying the persistence of racial disparities in birth outcomes continue to elude public health researchers. The findings presented in this study do not include strong patterns of association between lifetime cardiovascular risk profiles and racial differences in incidence of adverse birth outcomes despite sound theoretical plausibility. However, the ambiguity in our results underscores the need for more research that considers pre-pregnancy health status and biological pathways underlying racial disparities in birth outcomes. Future studies should aim for more precise measures and biological indicators of mechanism to improve our understanding of these outcomes.

## Additional file

Additional file 1: **Table S1.** Relationship between preconception cardiovascular risk factors and continuous birthweight, the Bogalusa Heart Study. (DOCX 14 kb)

## Abbreviations

BHS: Bogalusa Heart Study
BMI: Body mass index
HDL: High-density lipoprotein
LBW: Low birthweight
LDL: Low-density lipoprotein
LGA: Large-for-gestational-age
OR: Odds ratio
PTB: Preterm birth
SGA: Small-for-gestational-age
SSN: Social Security Number
