# Supplementary material for: Pre-pregnancy cardiovascular risk factors and racial disparities in birth outcomes: the Bogalusa Heart Study
Source: BMC Pregnancy Childbirth. 2018 Aug 20;18:339. doi: 10.1186/s12884-018-1959-y (PMC6102890; doi:10.1186/s12884-018-1959-y)
Supplement: Supplementary file 1 — Table S1. Relationship between preconception cardiovascular risk factors and continuous birthweight, the Bogalusa Heart Study. (DOCX 14 kb) [file 12884_2018_1959_MOESM1_ESM.docx]

| Table S1. Relationship between preconception cardiovascular risk factors and continuous birthweight, the Bogalusa Heart Study | | | | | | | | | | | | | | | | | | | |
| --- | --- | --- | --- | --- | --- | --- | --- | --- | --- | --- | --- | --- | --- | --- | --- | --- | --- | --- | --- |
|  |  | white | | | | | | |  | black | | | | | | |  |  |  |
|  |  | unadjusted | | |  | adjusted** | | |  | unadjusted | | |  | adjusted** | | |  | P for interaction | |
|  |  | β | se(β) | p |  | β | se(β) | p |  | β | se(β) | p |  | β | se(β) | p |  | unadjusted | adjusted** |
| systolic BP |  | 2.00 | 1.27 | 0.12 |  | 5.47 | 1.57 | 0.00 |  | 4.30 | 1.63 | 0.00 |  | 0.83 | 2.10 | 0.69 |  | 0.26 | 0.08 |
| diastolic BP |  | 4.70 | 1.40 | 0.00 |  | -2.67 | 1.75 | 0.13 |  | 3.30 | 1.73 | 0.06 |  | -1.53 | 2.21 | 0.49 |  | 0.52 | 0.89 |
| cholesterol* |  | -0.01 | 0.45 | 0.98 |  | -0.27 | 0.47 | 0.57 |  | 0.40 | 0.61 | 0.51 |  | 0.42 | 0.66 | 0.53 |  | 0.58 | 0.54 |
| triglycerides* |  | 0.26 | 0.26 | 0.33 |  | -0.23 | 0.29 | 0.43 |  | 0.51 | 0.65 | 0.43 |  | -1.17 | 0.75 | 0.12 |  | 0.26 | 0.08 |
| LDL* |  | 0.03 | 0.49 | 0.95 |  | -0.20 | 0.52 | 0.70 |  | 0.61 | 0.68 | 0.37 |  | 0.99 | 0.75 | 0.19 |  | 0.48 | 0.31 |
| HDL* |  | -0.45 | 0.84 | 0.59 |  | 0.36 | 0.93 | 0.70 |  | 0.26 | 1.21 | 0.83 |  | -1.07 | 1.40 | 0.45 |  | 0.63 | 0.60 |
| glucose* |  | -0.18 | 0.96 | 0.85 |  | 0.03 | 0.98 | 0.98 |  | 2.94 | 1.14 | 0.01 |  | 2.86 | 1.22 | 0.02 |  | 0.03 | 0.04 |
| insulin* |  | 3.32 | 2.38 | 0.16 |  | -0.60 | 2.78 | 0.83 |  | 0.40 | 2.02 | 0.84 |  | -1.97 | 2.19 | 0.37 |  | 0.35 | 0.27 |
| *fasted |  |  |  |  |  |  |  |  |  |  |  |  |  |  |  |  |  |  |  |
| ** adjusted for BMI, time since screening, maternal age, year of mother's birth, smoking, parity, maternal education, Kotelchuck prenatal care | | | | | | | | | | | | | | | | | | | |
